# Solving Molecular Docking Problems with Multi-Objective Metaheuristics

**DOI:** 10.3390/molecules200610154

**Published:** 2015-06-02

**Authors:** María Jesús García-Godoy, Esteban López-Camacho, José García-Nieto, Antonio J. Nebro, José F. Aldana-Montes

**Affiliations:** Khaos Research Group, Departament of Computer Sciences, University of Málaga (UMA), ETSI Informática, Campus de Teatinos, Málaga 29071, Spain; E-Mails: mjgarciag@lcc.uma.es (M.J.G.-G.); esteban@lcc.uma.es (E.L.-C.); jnieto@lcc.uma.es (J.G.-N.); antonio@lcc.uma.es (A.J.N.)

**Keywords:** molecular docking, multi-objective optimization, nature-inspired metaheuristics, algorithm comparison

## Abstract

Molecular docking is a hard optimization problem that has been tackled in the past with metaheuristics, demonstrating new and challenging results when looking for one objective: the minimum binding energy. However, only a few papers can be found in the literature that deal with this problem by means of a multi-objective approach, and no experimental comparisons have been made in order to clarify which of them has the best overall performance. In this paper, we use and compare, for the first time, a set of representative multi-objective optimization algorithms applied to solve complex molecular docking problems. The approach followed is focused on optimizing the intermolecular and intramolecular energies as two main objectives to minimize. Specifically, these algorithms are: two variants of the non-dominated sorting genetic algorithm II (NSGA-II), speed modulation multi-objective particle swarm optimization (SMPSO), third evolution step of generalized differential evolution (GDE3), multi-objective evolutionary algorithm based on decomposition (MOEA/D) and S-metric evolutionary multi-objective optimization (SMS-EMOA). We assess the performance of the algorithms by applying quality indicators intended to measure convergence and the diversity of the generated Pareto front approximations. We carry out a comparison with another reference mono-objective algorithm in the problem domain (Lamarckian genetic algorithm (LGA) provided by the AutoDock tool). Furthermore, the ligand binding site and molecular interactions of computed solutions are analyzed, showing promising results for the multi-objective approaches. In addition, a case study of application for aeroplysinin-1 is performed, showing the effectiveness of our multi-objective approach in drug discovery.

## 1. Introduction

Molecular docking is a hard optimization problem, which consists of predicting the orientation of a small molecule (ligand) to a receptor (macromolecule) when they are bound to each other to form an energetically-stable complex. The main goal is to find an optimal conformation between the ligand and receptor with a minimum binding energy, which is evaluated by an energy function. This problem has been tackled in the past with metaheuristics and other nature-inspired computational methods, demonstrating new and challenging results when looking for the minimum binding energy [1]. However, only a few papers can be found in the literature that deal with this problem by means of multi-objective approaches. The first attempt was carried out in 2006 by Oduguwa *et al*. [2], in which three evolutionary multi-objective optimization algorithms (non-dominated sorting genetic algorithm II (NSGA-II), the Pareto Archived Evolution Strategy algorithm (PAES) and the Strength Pareto Evolutionary algorithm (SPEA)) were evaluated on three molecular complexes. Grosdidier *et al*. [3] proposed a new hybrid evolutionary algorithm for docking (EADock), which was interfaced with the CHARMM package. In 2008, Janson *et al*. [4] designed a parallel multi-objective optimization algorithm based on PSO (Particle Swarm Optimization) using AutoDock energy function Version 3.05, called ClustMPSO, that used K-means to guide the migration strategy when dealing with six molecular complexes. Furthermore, in 2008, Boisson *et al*. [5] implemented a parallel evolutionary bi-objective model using ParadisEO platform and GOLD docking software for the docking of six instances. Recently, Sandoval-Perez *et al*. [6] used the implementation of NSGA-II provided by the jMetal framework [7] to optimize bound and non-bound energy terms as objectives applied to four docking instances. These publications, although proposing different approaches, performed only limited comparisons with other current multi-/single-objective techniques. Furthermore, a low number of molecular instances were used in these studies, and their complexities were also moderate in terms of flexibility. In the area of ligand design, there have been several studies that apply the multi-objective approach. Sanchez-Faddeev *et al*. [8] proposed a bi-objective optimization approach using the S-metric evolutionary multi-objective optimization (SMS-EMOA) to solve the problem of finding a peptide ligand. The results obtained show the possibility to design a peptide ligand of the *γ*1 isoform of the 14-3-3 protein with predicted selectivity over the *ɛ*1 isoform. Van der Horst *et al*. [9] used the multi-objective evolutionary algorithm (MOEA) for a *de novo* ligand design applied to the new adenosine receptor antagonists. The selection of the candidate A1 adenosine receptor antagonists was based on multiple criteria and several objectives, such as the high predicted affinity and the selectivity of the ligands for the receptors, and properties, like the ADMET (Absorption, Distribution, Metabolism, Elimination and Toxicity) score.

In this paper, our motivation is to compare and analyze the performance of six multi-objective metaheuristics when solving flexible molecular docking complexes. These algorithms are: the non-dominated sorting genetic algorithm II (NSGA-II) [10] and its steady-state version (ssNSGA-II) [11], speed modulation multi-objective particle swarm optimization (SMPSO) [12], third evolution step of generalized differential evolution (GDE3) [13], multi-objective evolutionary algorithm based on decomposition (MOEA/D) [14] and S-metric evolutionary multi-objective optimization (SMS-EMOA) [15]. We have selected these algorithms as they constitute a varied set of modern multi-objective techniques in the state-of-the-art, performing different learning procedures and, therefore, inducing different behaviors in terms of convergence, diversity and scalability. These algorithms have been shown to obtain successful performances on a wide variety of optimization problems [16,17]; however, they have not been previously used to solve the molecular docking problem, with the exception of the NSGA-II algorithm [6]. The application of these algorithms constitutes an additional contribution of this study. We use the implementation of these algorithms provided by jMetalCpp [18], the C++ version of the jMetal framework [7], to carry out a rigorous experimental study.

In order to assess the performance of these algorithms, we have used a benchmark of 11 complexes based on the HIV-protease receptors and ligands with different ligand sizes. This benchmark is a set of complexes used by [19] to prove that the energy function of AutoDock Version 4.2 allows one to perform docking simulations with flexible portions of the receptor. According to this, it is worth noting that it has been reported that the use of rigid macromolecules in docking simulations leads to inaccurate ligand binding estimations [20]. Therefore, we have opted to work with flexible macromolecule docking instances, instead of using rigid macromolecule instances, as is usually the case in the current literature [4,5]. The optimization approach followed in this study was proposed in [4], which has been focused on minimizing two contradictory objectives: intermolecular (*E_inter_*) and intramolecular energies (*E_intra_*). Moreover, the AutoDock energy function Version 4.2.3 [21] has been used in this study to evaluate the new solutions generated by the multi-objective solvers.

The resulting optimization strategy consists of a software package that integrates AutoDock + jMetal [18], in such a way that the algorithms available in jMetal can be used to optimize the energy function of AutoDock. The experimentation methodology performed in this paper consists of preparing the instances of the problem to be docked and computing a pre-fixed number of function evaluations. The results obtained have been statistically compared by considering two different quality indicators to measure the convergence and diversity of the obtained Pareto front approximations. Additionally, a comparison with the mono-objective approach has been performed, using as a base algorithm the Lamarckian genetic algorithm (LGA) [1], commonly used in AutoDock studies. Following this line of this comparison, an analysis of the ligand binding sites and the molecular interactions of two instances has been carried out with regard to the crystallographic structure.

The remainder of this study is organized as follows. Section 2 describes the molecular docking problem from a multi-objective formulation. A review of the metrics and algorithms studied is provided in Section 3. Section 4 describes the results obtained and the molecular docking analyses. Section 5 reports the experimentation methodology. Section 6 contains concluding remarks and future work.

## 2. Multi-Objective Molecular Docking

Before introducing the optimization strategy followed for molecular docking, a series of background concepts regarding the multi-objective optimization are formally defined in this section.

### 2.1. Multi-Objective Optimization

In current practice, many academic and industrial optimization problems need to address the resolution of a task where two or more objective functions are to be optimized, these all being equally important. A formal definition of a multi-objective optimization problem (MOP) is provided in the following formulation (Definition 1). Without loss of generality, we assume that minimization is the goal for all the objectives.

#### Definition 1 (MOP)

*Find a vector* ${\vec{x}}^{*}=[x_{1}^{*},x_{2}^{*},\ldots ,x_{n}^{*}]$* that satisfies the m inequality constraints g_i_* (*x⃗*) ≥ 0, *i* = 1, 2, . . . ,*m, the p equality constraints h_i_* (*x⃗*) = 0, *i* = 1, 2, . . . , *p and minimizes the vector function f⃗* (*x⃗*) = [*f*_1_(*x⃗*), *f*_2_(*x⃗*), . . . , *f_k_*(*x⃗*)]*^T^, where x⃗* = [*x*_1_, *x*_2_, . . . , *x_n_*]*^T^* *is the vector of decision variables.*

The set of all values satisfying the constraints defines the feasible region Ω, and any point *x⃗* ∈ Ω is a feasible solution. We seek the Pareto optima. More formally:

#### Definition 2 (Pareto optimality)**.**

A point x⃗^*^ ∈ Ω *is Pareto optimal if for every x⃗* ∈ Ω *and I* = {1, 2, . . . , *k*}*, either* ∀*_i∈I_* (*f_i_* (*x⃗*) = *f_i_*(x⃗^*^)) *or there is at least one i* ∈ *I, such that f_i_* (*x⃗*) > *f_i_* (x⃗^*^)*.*

This definition states that x⃗^*^ is Pareto optimal if no other feasible vector *x⃗* exists that would improve some criteria without causing a simultaneous worsening of at least one other criterion. Other important nomenclature associated with Pareto optimality is defined below:

#### Definition 3 (Pareto dominance)**.**

*A vector u⃗* = (*u*_1_, . . . , *u_k_*) *is said to dominate v⃗* = (*v*_1_, . . . , *v_k_*) *(denoted by u⃗* ≼ *v⃗) if and only if u⃗ is partially less than v⃗,* i.e.*,* ∀*i* ∈ {1, . . . , *k*} , *u_i_* ≤ *v_i_* ∧ ∃*i* ∈ {1, . . . , *k*} : *u_i_* *< v_i_*.

#### Definition 4 (Pareto optimal set)**.**

*For a given MOP f⃗*(*x⃗*)*, the Pareto optimal set is defined as* ℘^*^ = {*x⃗* ∈ Ω|¬∃*x⃗*′ ∈ Ω, *f⃗*(*x⃗*′) ≼ *f⃗*(*x⃗*)}*.*

#### Definition 5 (Pareto front)**.**

*For a given MOP f⃗*(*x⃗*) *and its Pareto optimal set* ℘^*^, the Pareto front is defined as ℘ℱ^*^ = {*f⃗*(*x⃗*), *x⃗* ∈ ℘^*^}*.*

Obtaining the Pareto front of a MOP is the main goal of multi-objective optimization. In theory, a Pareto front could contain a large number (or even infinitely many) points. In practice, a usable approximate solution will only contain a limited number of them; thus, an important goal is that they should be as close as possible to the exact Pareto front (convergence) and uniformly spread (diversity); otherwise, they would not be very useful to the decision maker. Closeness to the Pareto front ensures that we are dealing with optimal solutions, while a uniform spread of the solutions means that we have made a good exploration of the search space and no regions are left unexplored.

The use of Pareto optimality-based techniques means dealing with a set of non-dominated solutions, which requires some specific mechanisms to handle them. Additionally, this set must be diverse enough to cover the whole front. Although depending on the algorithm, there are many different issues to deal with: fitness function, diversity management and constraint handling mechanisms. During the iteration of any metaheuristic algorithm, there always exists a phase in which all of the solutions must be sorted to pick one (or more) of them. Examples of these phases are the selection and replacement mechanisms in EAs. In single-objective optimization, the fitness is a single (scalar) value, and thus, the sorting is done according to it. However, in the multi-objective domain, the fitness consists of a vector of values (one value per objective function), and as a consequence, the sorting is not straightforward.

### 2.2. Molecular Docking Optimization Strategy

In a multi-objective optimization problem, it is necessary to define two spaces: the decision and the objective spaces. The decision space involves all sets of solutions, and the objective space includes the fitness (scoring) values of the objectives to be optimized.

The main objective in the molecular docking problem is to find an optimized conformation between the ligand (*L*) and the receptor (*R*) that results in a minimum energy that binds to a particular protein of interest. A formal definition of the molecular docking problem is as follows [22]:

#### **Definition 6** (Docking optimization)**.**

*Let A and B be the ligand and protein molecule, respectively. Let f be a scoring (energy) function that ranks solutions with regards to binding energy, and let C be the conformational (decision) search space of all possible conformations (docking solutions) between A and B. Then, the docking problem is an optimization problem aimed at finding a x⃗* ∈ *C satisfying f*(*x⃗*) ≤ *f*(*y⃗*)∀*_y⃗_*_∈_*_C_* *(minimization).*

Despite great efforts in computational algorithmics, molecular docking remains a challenging problem generally considered to be NP-Hard (although, no formal proof exists, to the best of our knowledge) [22]. Therefore, to deal with a docking flexibility search space in an efficient manner, modern search techniques, such as metaheuristics, are highly recommendable. In this study, our aim is also to take advantage of the specific learning procedure induced by multi-objective (metaheuristics) approaches to tackle molecular docking.

##### Decision space

**Figure 1.**
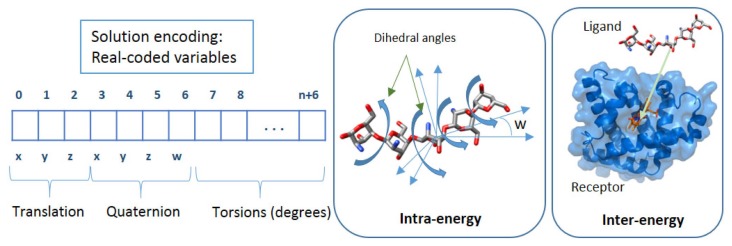
Solution encoding in AutoDock 4.2 and jMetal. The first three values (translation) are the coordinates of the center of rotation of the ligand. The next four values (quaternion) are the unit vector describing the direction of rigid body rotation (*x*, *y* and *z*) and the rotation of the angle degrees (*w*) that are applied. The rest of the values holds the torsion angles in degrees, *n* being the number of torsions of the ligand and also the receptor. Movements concerning the *E_inter_* and *E_intra_* calculation are also represented.

The interaction between ligand and receptor can be described by an energy function calculated through three components representing degrees of freedom: (1) the translation of the ligand molecule, involving the three axis values (*x*, *y*, *z*) in Cartesian coordinate space; (2) the ligand orientation, modeled as a four-variable quaternion, including the angle slope (*ω*); and (3) the flexibilities, represented by the free rotation of torsion (dihedral angles) of the ligand and side chains of the receptor. Therefore, each problem solution for AutoDock and jMetal (the tools we have used) is encoded by a real-value vector of 7 + *n* variables (illustrated in Figure 1), in which the first three values correspond to the ligand translation, the next four values correspond to the ligand and/or macromolecule orientation and the remaining *n* values are the ligand torsion dihedral angles. Nevertheless, in order to reduce the computational cost, a grid-based methodology has been implemented where the protein active site is embedded in a 3D rectangular grid, and at each point of the grid, the electrostatic interaction energy and the Van der Waals terms for each ligand atom type are pre-computed and stored, taking into account all of the protein atoms [23]. In this way, the protein contribution at any given point is obtained by tri-linear interpolation in each grid cell. This interpolation leads to a range of translation variables (*x*, *y*, *z*) of a 120-grid spacing point dimension. The values of these variables are delimited between the range of the coordinates of the grid space that has been set for each problem. In the case of orientation (quaternion) and torsion variables, they are measured in radians and encoded in the range of [−*π*, *π*].

##### Objective space

From a mono-objective optimization perspective, the objective to be optimized is the free energy of binding, which is calculated as shown in Equation (1). However, this goal can be formulated as a bi-objective optimization problem by taking into account the *E_inter_* and *E_intra_*, respectively. These energy terms are given by the AutoDock energy function [19] (see Equation (1)), being opposite from each other [4] and, therefore, giving rise to a multi-objective approach of this problem as follows:

- Objective 1: the *E_intra_* energy (Equation (2)) of the ligand and receptor is estimated by the difference between the bound and unbound states of the ligand and receptor.
- Objective 2: the *E_inter_* energy (Equation (3)) is estimated by the difference of the bound and unbound states of the ligand-macromolecule complex.
  (1) $$\Delta G=E_{intra}+E_{inter}+\Delta S_{conf}$$
  (2) $$E_{intra}=\left(Q_{bound}^{L-L}-Q_{unbound}^{L-L}\right)+\left(Q_{bound}^{R-R}-Q_{unbound}^{R-R}\right)$$
  (3) $$E_{inter}=\left(Q_{bound}^{R-L}-Q_{unbound}^{R-L}\right)$$

Each pair of energetic evaluation terms in *E_inter_* and *E_intra_* includes evaluations (*Q*) of dispersion/repulsion forces (*vdw*), hydrogen bonds (*hbond*), electrostatics (*elec*) and desolvation (*sol*). Weights *W_vdw_*, *W_hbond_*, *W_conf_*, *W_elec_* and *W_sol_* of Equation (4) are constants for Van der Waals, hydrogen bonds, torsional forces, electrostatic interactions and desolvation, respectively. *r_ij_* represents the interatomic distance; *A_ij_* and *B_ij_* in the first term are Lennard–Jones parameters taken from the Amber force field. Similarly, *C_ij_* and *D_ij_* in the second term are Lennard–Jones parameters for the maximum well depth of potential energies between two atoms, and *E*(*t*) represents the angle-dependent directionality. The third term uses a Coulomb approach for electrostatics. Finally, the fourth term is calculated from the volume (*V* ) of the atoms that are surrounding a given atom weighted by *S* and an exponential term, which involves atom distances. An extended explanation of all of these variables can be found in [19].

(4) $$Q=W_{vdw}\sum_{i,j}\left(\frac{A_{ij}}{r_{ij}^{12}}-\frac{B_{ij}}{r_{ij}^{6}}\right)+W_{hbond}\sum_{i,j}E(t)\;\left(\frac{C_{ij}}{r_{ij}^{12}}-\frac{D_{ij}}{r_{ij}^{10}}\right)+W_{elec}\sum_{i,j}\frac{q_{i}q_{j}}{ɛ(r_{ij})r_{ij}}+W_{sol}\sum_{i,j}(S_{i}V_{j}+S_{j}V_{i})e^{\left(-r_{ij}^{2}/2\sigma^{2}\right)}$$

## 3. Metrics and Algorithms

Once the optimal docking problem has been defined, this section provides a summarized description of selected multi-objective approaches for this study. Before, the scoring metrics used to evaluate these algorithms are included.

### 3.1. Metrics

Contrary to single/mono-objective optimization, where assessing the performance of a metaheuristic mainly requires observing the best value yielded by an algorithm (*i.e.*, the lower the better, in the case of minimization), in multi-objective optimization, this is not applicable. Instead, an approximation set to the optimal Pareto front of the problem is computed. Two properties are usually required: convergence and a uniform diversity. A number of quality indicators for measuring these two criteria have been proposed in the literature [17].

In this work, we have concentrated on two quality indicators: the hyper-volume (*I_HV_* ) and unary additive epsilon indicators (*I_ɛ_*_+_) [17]. The first indicator takes into account both convergence and diversity, whereas the second one (*I_ɛ_*_+_) is used more to validate the convergence behavior.

**Figure 2.**
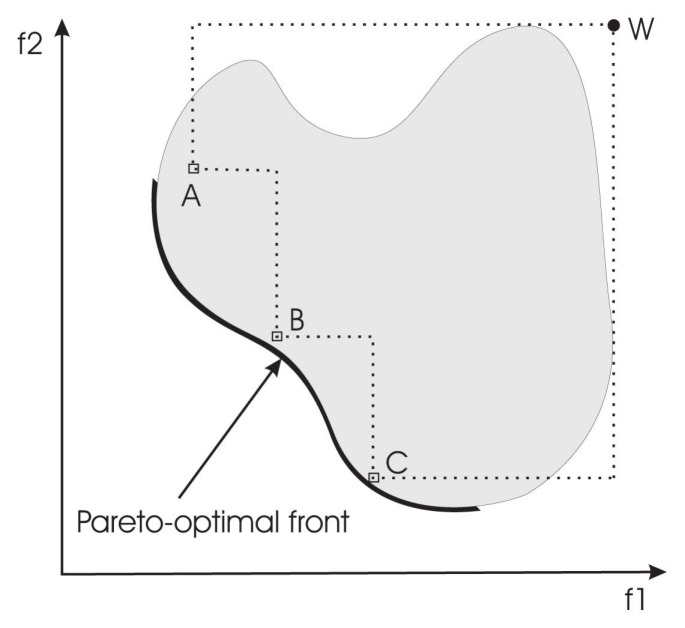
The hyper-volume (delimited by the dotted line) enclosed by the non-dominated solutions. *A*, *B*, and *C* are non-dominated solutions in a set *Q* = {*A*,*B*,*C*}. For each solution *i* ∈ *Q*, a hypercube *v_i_* is constructed with a reference point *W* and the solution *i* as the diagonal corners of the hypercube. The reference point *W* can be found by constructing a vector of worst objective function values.

- *I_HV_* : This indicator calculates the *n*-dimensional space covered by members of a non-dominated set of solutions *Q*, e.g., the region enclosed by the discontinuous line in Figure 2, *Q* = {*A*,*B*,*C*}, for problems where all objectives are to be minimized. Mathematically, for each solution *i* ∈ *Q*, a hypercube *v_i_* is constructed with a reference point *W* and the solution *i* as the diagonal corners of the hypercube. The reference point can simply be found by constructing a vector of worst objective function values. Thereafter, a union of all hypercubes is found, and its hyper-volume (*I_HV_* ) is calculated:
  (5) $$I_{HV}=\text{volume }\left(\overset{|Q|}{\underset{i=1}{\cup }}v_{i}\right)$$
  Solution fronts with larger values of *I_HV_* are desirable.
- *I_ɛ_*_+_: Given a computed front for a problem, *A*, this indicator is a measure of the smallest distance one would need to translate every solution in *A*, so that it dominates the optimal Pareto front of this problem. More formally, given $\vec{z^{1}}=(z_{1}^{1},\ldots ,z_{n}^{1})$ and $\vec{z^{2}}=(z_{1}^{2},\ldots ,z_{n}^{2})$, where *n* is the number of objectives:
  (6) $$I_{ɛ+}^{1}(A)=inf_{ɛ\in \mathbb{R}}\left\{\forall \vec{z^{2}}\in PF^{*}\exists \vec{z^{1}}\in A:\vec{z^{1}}{≺}_{ɛ}\vec{z^{2}}\right\}$$
  where $\vec{z^{1}}{≺}_{ɛ}\vec{z^{2}}$ if and only if $\forall 1\leq i\leq n:z_{i}^{1}<ɛ+z_{i}^{2}$. In this case, solution fronts with lower values of *I_ɛ_*_+_ are desirable.

#### 3.2. Algorithms

We have selected these six algorithms, which are representative of the state-of-the-art in the multi-objective optimization field. These techniques consists of: the most widely-used MO algorithm in the field (NSGA-II) [10], a steady-state variant of it (ssNSGA-II) [11], a swarm intelligence-based approach (SMPSO) [12], a solver based in differential evolution (GDE3) [13], an algorithm representative of decomposition-based techniques (MOEA/D) [14] and an algorithm representing indicator-based metaheuristics (SMS-EMOA) [15]. A brief description of these techniques is given next:

**NSGA-II:** NSGA-II [10] is the most widely-used multi-objective optimization algorithm. It is a genetic algorithm based on obtaining a new individual from the original population by applying the typical genetic operators (selection, crossover and mutation). A ranking procedure is applied to promote convergence, while a density estimator (the crowding distance) is used to enhance the diversity of the set of found solutions.
**ssNSGA-II:** The steady-state version of the NSGA-II was presented in [11]. This study showed that ssNSGA-II outperformed NSGA-II in a set of benchmark problems, although at the cost of increasing the running time with respect to the original algorithm.
**SMPSO:** SMPSO [12] is a multi-objective particle swarm optimization algorithm whose main characteristic is the use of a strategy to limit the velocity of the particles, in order to allow new effective particle positions to be produced in those cases where the velocity becomes too high. Furthermore, SMPSO includes the polynomial mutation as the turbulence factor and an external archive that stores the non-dominated solutions found during the search.
**GDE3:** The generalized differential evolution (GDE) algorithm [13] is based on NSGA-II, but the crossover and mutation variation operators are changed to use the differential evolution operator; concretely, it uses the rand/1/bin variant. Another difference is that GDE3 modifies the crowding distance of NSGA-II to generate a better distributed set of solutions.
**MOEA/D:** MOEA/D [14] is based on decomposing a multi-objective optimization problem into a number of scalar optimization subproblems, which are optimized simultaneously, only using information from their neighboring subproblems. We have used the variant MOEA/D-DE [24], which applies differential evolution instead of the genetic crossover and mutation operators used in the original algorithm. This algorithm also applies a mutation operator to the solutions.
**SMS-EMOA:** When comparing the results of evolutionary multi-objective optimization algorithms (EMOA), the hyper-volume measure quality (also called the S-metric) can be applied. The S-metric selection steady-state EMOA [15] uses a selection operator based on the hyper-volume measure combined with the concept of non-dominated sorting. Its main feature lies in making its population evolve to a well-distributed set of solutions, in order to focus on interesting regions of the Pareto front, at the cost of the high computing time of computing the hyper-volume. To cope with this issue, we have incorporated the WFG hyper-volume [25] into SMS-EMOA, which is very efficient in the case of bi-objective problems, as the one we are solving. In this approach, the reference point to measure the hyper-volume contribution is based on adding a constant offset to the maximum value of each objective.

## 4. Results and Discussion

In this section, a series of analyses in terms of algorithmic performance comparisons, mono/multi-objective approaches, ligand binding site and molecular interactions are reported. Furthermore, an actual case study based on aeroplysinin-1 compounds for a drug design application is described.

### 4.1. Performance Comparisons

Table 1 shows the median and interquartile range of the computed solutions for *I_HV_* and *I_ɛ_*_+_ quality indicators for the set of 11 docking instances and the six algorithms being compared: NSGA-II, ssNSGA-II, SMPSO, GDE3, MOEA/D and SMS-EMOA. In the case of *I_HV_*, the higher the median value, the better the result, whereas for *I_ɛ_*_+_, the lower the numerical values, the higher the quality of solutions.

The hyper-volume is a quality indicator that takes into account both convergence and diversity. According to the reported results, SMPSO achieves the best *I_HV_* values in seven out of the eleven considered problems; GDE3 is the second best performing technique. The *I_ɛ_*_+_ provides a measure of convergence, and the figures in Table 1 confirm SMPSO as the metaheuristic providing the best overall performance.

In order to present these results with statistical confidence (in this study, *α* = 0.05), a series of non-parametric statistical tests have been applied (in several cases, the distributions of the results did not follow the conditions of normality and homoscedasticity [26]). Therefore, the analyses and comparisons focus on the entire distribution of each of the two metrics studied. Specifically, Friedman’s ranking and Holm’s *post hoc* multi-comparison tests [26] have been applied in order to know which algorithms are statistically worse than the control one (the algorithm with the best ranking). This combination of Friedman’s ranking and *post hoc* Holm’s test has been shown to offer a well-balanced statistical framework, mostly on non-parametric distribution results [26–28]. Friedman’s test establishes a first ranking of algorithms in order to choose a control sample (best ranked algorithm), which is used in Holm’s *post hoc* test for multi-comparison. This statistical framework has been recommended in the specialized literature [26–28], as it is well adapted to compare stochastic based methods, such as metaheuristics. In this regard, as shown in Table 2, GDE3 reaches the best ranking value (Friedman) with 1.81 for the HV indicator, followed by SMPSO, MOEA/D, SMS-EMOA, NSGA-II and ssNSGA-II. Therefore, GDE3 is established as the control algorithm for HV in the *post hoc* Holm test, which is compared with the remaining algorithms. The adjusted *p*-values (*Holm_Ap_* in Table 2) resulting from these comparisons are, for the last three algorithms (SMS-EMOA, NSGA-II and ssNSGA-II), lower than the confidence level, meaning that GDE3 is statistically better than these algorithms. In the case of *I_ɛ_*_+_, SMPSO is better ranked than MOEA/D and GDE3, although without statistical differences in these cases. SMPSO is statistically better than NSGA-II, SMS-EMOA and ssNSGA-II.

Broadly, summing up all ranking positions (as shown in the right-hand column of Table 2), we can observe that SMPSO shows the overall best balance for the two quality indicators. In addition, this algorithm obtained statistically better results than NSGA-II, SMS-EMOA and ssNSGA-II. GDE3 obtained the second best performance, followed by MOEA/D.

**Figure 3.**
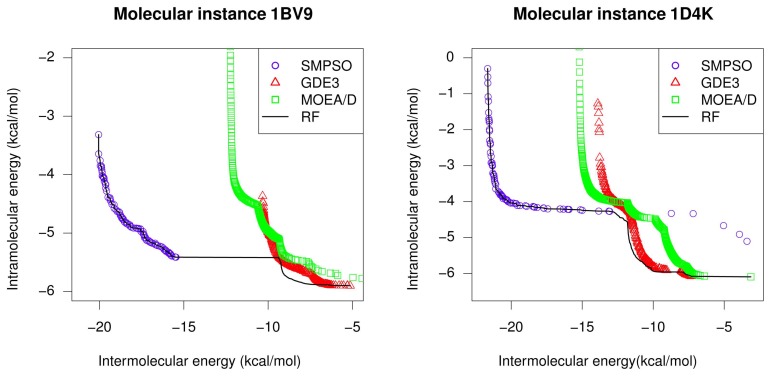
Reference fronts and best hyper-volumes from SMPSO, GDE3 and MOEA/D for instances 1BV9 and 1D4K.

These results are graphically supported by two examples included in Figure 3, where the fronts having the highest hyper-volume values obtained by SMPSO, GDE3 and MOEA/D for instances 1BV9 and 1D4K are plotted against the reference front (RF black curve). In this figure, it is easily observable that SMPSO always obtains solutions in regions of the reference front where GDE3 and MOEA/D do not converge. These last two algorithms show a good spread of solutions in their Pareto front approximations, but with a limited convergence to only one region of the reference front. An interesting observation in this sense is that, for all studied molecular instances, SMPSO converges to the region biased towards the *E_inter_* objective (left-hand side of reference front), whereas GDE3 and MOEA/D generate their fronts of non-dominated solutions in a different region to the ones of SMPSO, therefore focused in this case on the intramolecular energy optimization (right-hand side of reference front).

We can state that the specific learning procedures induced by SMPSO and GDE3 lead these algorithms to search in different regions of the problem landscape, hence generating solutions in complementary parts of the reference front.

### 4.2. Comparison with a Mono-Objective Approach

To analyze the benefit of using the multi-objective formulation of the molecular docking from the decision-maker’s (*i.e.*, a biologist) point of view, we have also solved the problem instances with the mono-objective LGA technique provided by AutoDock 4.2. In order to allow comparisons, we have used Equation (1) to recalculate the mono-objective fitness values of the Pareto front approximations yielded by SMPSO.

Table 3 shows a mono-objective comparison of the best solutions obtained (out of 30 independent runs) from both the SMPSO algorithm and the LGA for all of the instances. In general, we can observe that SMPSO outperforms LGA for almost all problem instances, but for 1HTF and 1HTG, although showing close binding energies in these cases. These results can be explained by the small size of the ligand in the case of the 1HTF complex. As was reported in [19,29], the experiments that involve small ligands and flexibility in the ARG-8 side chains of the macromolecule increase the size of the conformational space. This can explain the final binding energy results obtained by the SMPSO in comparison with the LGA algorithm. In the case of the 1HTG complex, this instance includes a larger HIV-protease inhibitor. Despite there being a difference between the results obtained by the SMPSO and the LGA algorithm, the difference of the final binding energy values obtained is smaller than the rest of the instances (except 1HTF). These results obtained can be explained due to the stochastic component of the algorithms used. Furthermore, it is worth mentioning that the 1HTG computed ligand conformation returned by the SMPSO is bound to the active site. Therefore, we have to note that even using the mono-objective formulation (as done in AutoDock), the multi-objective general purpose approach by SMPSO is able to provide experts with more optimized solutions than LGA, this last technique being specifically designed for the molecular docking problem.

**Figure 4.**
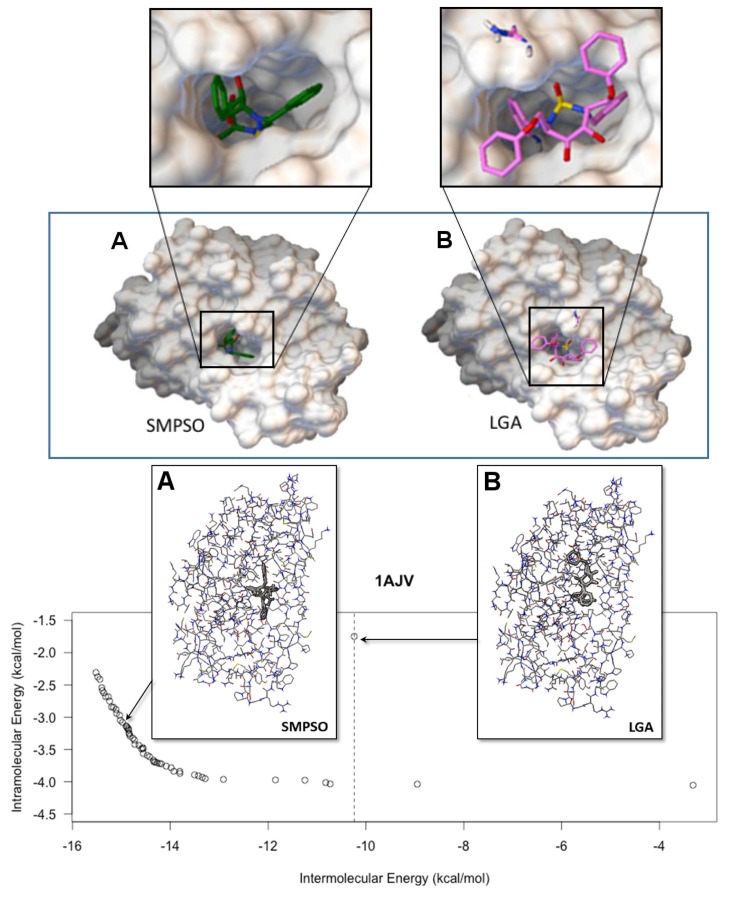
Front having the best hyper-volume value from SMPSO with regards to instance 1AJV. The point on the dashed vertical line is the best solution found by LGA, the mono-objective algorithm from AutoDock. All solutions placed on the left-hand side dominate that of LGA for the two optimized objectives. The macromolecule-ligand complexes resulted from the SMPSO and the LGA algorithms are represented in A and B, respectively.

Figure 4 shows the front that has the highest hyper-volume value obtained by SMPSO for the instance 1AJV. This instance involves a cyclic urea inhibitor and an HIV-protease macromolecule, a complex problem given the ligand features. The best LGA solution is presented as a point with a dashed vertical line, this solution being the sum of the *E_inter_* and *E_intra_* resulting from the LGA algorithm. The front solutions to the left of the dashed vertical dominate the best LGA solution, while those to the right side have better energy values in the *E_intra_* objective. In this regard, the selection of one energy or another depends on the biology expert. For example, the biologist can be interested in either a solution with a smaller *E_intra_* and a more stable inhibitor conformation or a solution with a smaller *E_inter_* and a more stable ligand-macromolecule complex. Therefore, considering the mono-objective fitness function, the solutions obtained from the SMPSO generally show a more stable docking conformation than the ones of LGA.

In Figure 4, the inhibitor docked to the HIV-protease macromolecule of the instance 1AJV is also observable. The ligand-macromolecule complexes of this figure show the inhibitor conformation resulting from the SMPSO solution and the inhibitor conformation from LGA. The first complex is energetically more stable than the second one given the energy results obtained; the binding energy for the first one corresponds to −11.57 kcal/mol and the second one to −7.26 kcal/mol. Both inhibitors are docked to the active site of the HIV-protease macromolecule, but the SMPSO inhibitor conformation has a better docking position.

### 4.3. Analysis on Ligand Binding Site and Molecular Interactions

**Figure 5.**
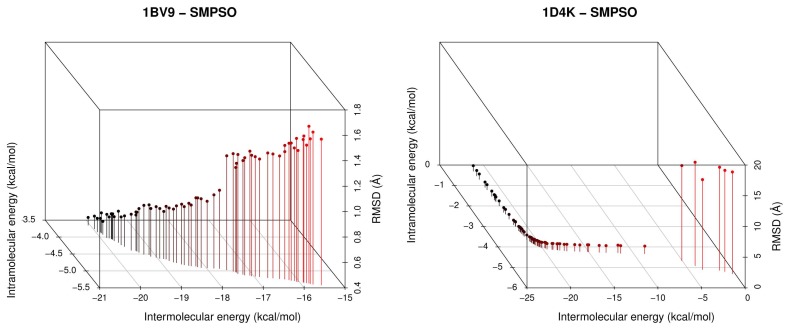
Non-dominated solution fronts of SMPSO for instances 1BV9 and 1D4K. The vertical bars represent the RMSD values for each solution. The solutions with better RMSD values are darker, that is the lower the RMSD, the better the docking solution is.

In addition to the analysis done in the previous subsection, we have carried out a new comparison of the SMPSO and LGA algorithms, but in terms of the resulting RMSD (Å) values. For this purpose, we have focused on instances 1DK4 and 1BV9 in this analysis, in order to extend our previous analyses concerning reference fronts in Figure 3. Figure 5 shows the reference fronts generated from all non-dominated solutions obtained through 30 runs of the SMPSO algorithm for instances 1DK4 and 1BV9. The colored vertical bars depict the RMSD values obtained for each solution. These RMSDs were calculated according to the average distance of the atomic coordinates between computed and reference ligands. As shown in this figure, the bars that represent better RMSD results (lower distance between computed and reference ligands) are darker than those bars that depict worse RMSD results. According to the solutions that are shown in Figure 5 for instances 1BV9 and 1D4K, the results with worse RMSD values correspond to solutions with higher *E_inter_* and lower *E_intra_*. In contrast, those solutions with better RMSD show lower *E_inter_* and higher *E_intra_* values.

**Figure 6.**
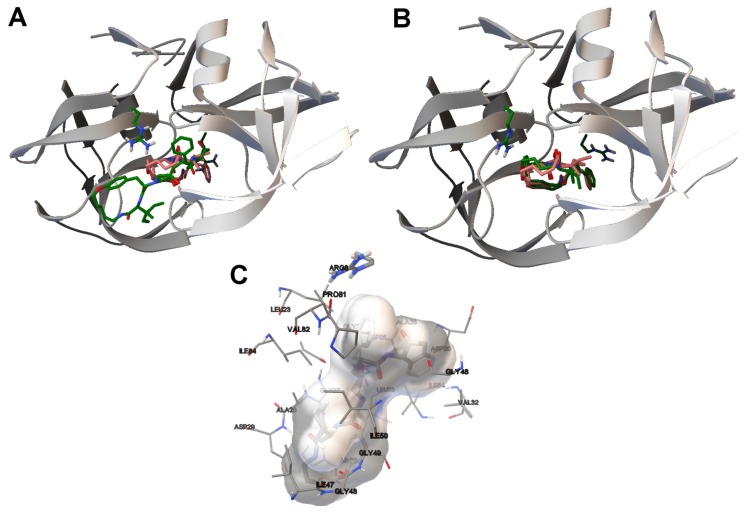
(**A**,**B**) The ligand conformation for instance 1D4K that has been generated by the LGA and SMPSO algorithms, respectively. The computed ligand conformations are in green and the reference ligand in orange. (**A**,**B**) show the HIV-protease in 3D, in grey; (**C**) The molecular interactions between the ligand conformation returned by the SMPSO algorithm and the receptor. The H-bonds are represented by green spheres. The amino acids closest to the computed ligand have been labeled.

**Figure 7.**
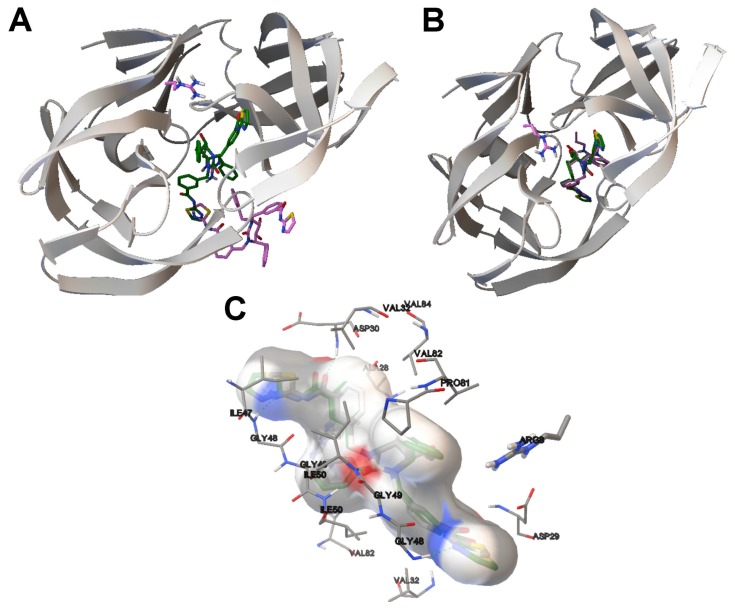
(**A**,**B**) The ligand conformations for instance 1BV9 that have been generated by the LGA and SMPSO algorithms. The computed ligand conformations are in purple and the reference ligand in green. (**A**,**B**) show the HIV-protease in 3D, colored in grey; (**C**) The molecular interactions between the ligand conformation returned by the SMPSO algorithm and the receptor. The H-bonds are represented by green spheres. The amino acids closest to the computed ligand have been labeled.

In order to perform an analysis based on the ligand binding site of the 1DK4 and 1BV9 instances and the molecular interactions, we have selected a solution for each instance from the non-dominated solution fronts of Figure 5. The criteria followed in choosing these two solutions was achieving a balance between the RMSDs, the *E_inter_* and *E_intra_*. For the LGA algorithm, we have selected the two best solutions for 1D4K and 1BV9, obtained in terms of the binding energy. The ligand conformations from the SMPSO and LGA algorithms and the reference ligand are compared in Figures 6 and 7.

In Figure 6, image (A) shows the best energy solution (the ligand in green) obtained by the LGA algorithm for instance 1D4K and the reference ligand (in orange). Image (B) shows the solution selected from the non-dominated solution fronts and the reference ligand. As shown, the inhibitor in (B) has a better conformation than in (A) given that the ligand conformation obtained by the SMPSO is closer to the tunnel-shaped active site of the HIV-protease receptor, this portion being very similar to the reference ligand. The RMSD scores of the ligand conformations by the LGA and SMPSO algorithms are 6.03 Å and 0.79 Å, respectively. Image (C) shows the H-bonding interactions between the ligand conformation returned by the SMPSO and the receptor. The ASP29 interacts with the ligand through a hydrogen bond in the same way as has been reported for the reference ligand, in line with other authors’ conclusions [30] with respect to the resulting conformations of SMPSO solutions found in the literature.

In Figure 7, (A) and (B) show the best energy solution by the LGA and the solution selected from the non-dominated solution fronts for instance 1BV9. As in the 1D4K example, the best ligand conformation corresponds to the solution returned by the SMPSO. The ligand conformation computed by the SMPSO is better positioned in the active site of the HIV-protease and, therefore, with respect to the reference ligand. The RMSD scores obtained for the LGA and the SMPSO are 8.79 Å and 0.59 Å. Figure (C) shows the H-bonds between the inhibitor and the receptor. The ASP30, GLY48 and ILE50 interact with the ligand through a hydrogen bond. In fact, it was shown that these amino acids are involved in the interaction between the reference ligand and the receptor [31].

### 4.4. Application of Multi-Objective Docking in Drug Discovery: A Use Case Based on the Aeroplysinin-1 Compound and EGFR

In this section, we move a step forward by presenting a case study applying our multi-objective docking approach in drug discovery. We have selected three non-dominated solutions (encoding docking conformations) from several executions of SMPSO, as it has been the best performing algorithm in our previous comparisons. Therefore, once we have tested SMPSO on an academic benchmark of chemical compounds related to the HIV-protease (which was used for AutoDock 4 studies to test the new force field), we apply this technique here in the scope of a real study case with the aeroplysinin-1 and EGFR (epidermal growth factor receptor) for drug discovery.

It is worth mentioning that aeroplysinin-1 is a bromo-compound produced by *Verongia* sponges as a chemical defense to protect them from bacterial pathogens, such as *Staphylococcus albus*, *Bacillus cereus* and *Bacillus subtilis* [32,33]. Aeroplysinin-1 has been extracted *in vitro* [34], and several analogues have been synthesized from this compound given its inhibitory activity against tyrosine kinases [35]. As growth factors, like EGF (epidermal growth factor) and VEGF (vascular epidermal growth factor), are involved in the regulation of the cell growth and proliferation, the targets of these factors are mostly receptors with tyrosine kinase activity (TKA), so aeroplysinin-1 is a candidate TKA receptor inhibitor for testing *in silico* and *in vitro*. Therefore, anti-tumoral action has been reported in several *in vitro* studies in which aeroplysinin-1 has a cytotoxic effect against tumoral cells from different tissues [36] and also an anti-angiogenic effect in the previous phases of the angiogenesis process [37]. Other studies have reported that aeroplysin-1 inhibits the kinase activity of the EGFR and induces the accumulation of this receptor in human breast tumoral cells [38].

Accordingly, we have selected a use case based on a study *in silico* with the aeroplysinin-1 compound and the cellular ecto- and intra-domains of the EGFR using the proposed multi-objective approach in this paper. With this use case, we attempt to understand how aeroplysinin-1 interferes in the kinase activity of the EGFR given the *in vitro* studies that have been performed with this compound. In this regard, the multi-objective approach presented in this paper provides the expert with a tool for assisting them with the selection of specific docking solutions according to the weight of the *E_inter_* and *E_intra_*. The selection of a specific docking solution would depend on the drug discovery problems of the expert.

In order to carry out the docking studies with the multi-objective technique presented, we obtained the crystallographic structures for the EGFR from the PDB database. For the EGFR intradomain of *Homo sapiens*, 1M17 has been used. This crystallographic structure presents the tyrosine domain kinase that includes residues 671 to 998 and the known anti-tumoral erlotinib drug (OSI-774, CP-358,774, TarcevaTM), which is an EGFR kinase-specific inhibitor. The 1YY9 crystal structure of *Homo sapiens*, which includes residues 25 to 642, has been selected for the EGFR ecto-domains. The aeroplysinin-1 (+) isomer was drawn using the ACD/ChemSketch software [39] given that the aeroplysinin-1 crystallographic structure has not been found.

Aeroplysinin-1 docking instance preparation: firstly, we used ADT to detect the four rotatable bounds, add the partial atomic charges and calculate the AutoDock atom types. For the preparation of the EGFR ecto- and endo-domains, the Chimera UCSF software was used to separate the intracellular kinase domain and the ecto-domains from their respective co-crystallized ligands and to remove all crystallographic molecules that are not involved in the ligand-receptor interaction, such as *N*-acetyl-glucosamine, alpha-mannose, *etc*. ADT was also used to add polar hydrogens and partial charges to the two EGFR macromolecules. To calculate the map, we established a grid that included all of the domains of both EGFR macromolecules with a grid spacing of 0.375 Å. The resulting files were used as inputs to run AutoGrid and AutoDock with jMetal. The SMPSO algorithm has been set with a population of 150 individuals, as done in our previous benchmark experiments. In this case, as we are dealing with real case studies, we set our algorithm with a larger number of energy evaluations (25,000,000) per run (30 independent runs), hence looking for optimized solutions with exhaustive experimentation.

**Figure 8.**
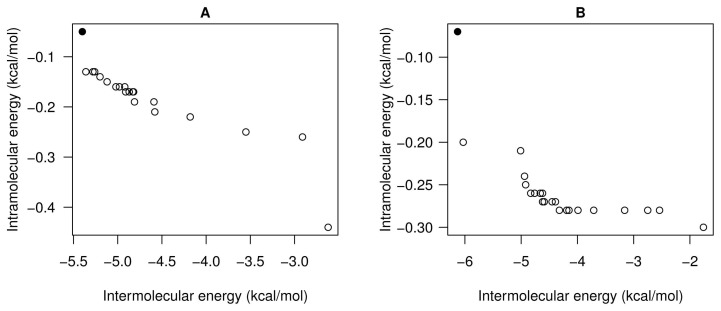
(**A**,**B**) show the SMPSO fronts of non-dominated solutions of Runs 7 and 23 (out of 30) for the aeroplysinin-1 docking instance. The black points are the solutions selected from the set of solutions of each run. The final binding energy of the black point at left side of the front corresponds to −4.21 kcal/mol. The *E_inter_* and *E_intra_* values are −5.4 and −0.05 kcal/mol, respectively. The final binding energy of the black point of the right boxplot corresponds to −4.93 kcal/mol. The *E_inter_* and *E_intra_* equal −6.13 and −0.07 kcal/mol, respectively.

**Figure 9.**
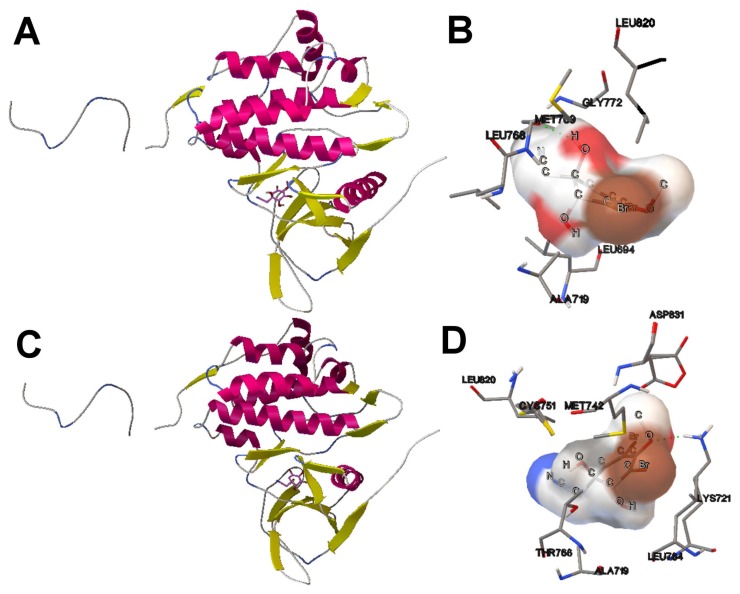
(**A**,**C**) The tridimensional structure of the EGFR kinase domain with the alpha-helix structure in pink, the beta-strands in yellow and aeroplysinin-1 bound to the cleft between the NH2 and COOH terminal lobes. These aeroplysinin-1 conformations correspond to the results obtained by the SMPSO algorithm (Runs 7 and 23); (**B**,**D**) The molecular interactions between the aeroplysinin-1 compound and the EGFR receptor. The H-bonds are represented in green spheres. The amino acids of the interaction site and the ligand atoms have been labeled.

Figure 8 shows the set of non-dominated solutions from two independent SMPSO runs of the EGFR kinase domain and the aeroplysinin-1 compound. In this instance, we have focused on those solutions (see the black points in Figure 8) with more negative *E_inter_*. The values of *E_inter_* of the solutions from Runs 7 and 23 are −5.4 and −6.13 kcal/mol, respectively. The *E_inter_* represents the binding affinity between the aeroplysinin-1 compound and the EGFR kinase domain. In this case, it is expected that the aeroplysinin-1 adopts an energetically-stable conformation to the cleft between the amino-terminal and carboxyl-terminal lobes of the EGFR domain kinase, as has been reported in the literature with other compounds, such as the Erlotinib (a 4-anilinoquinazoline inhibitor), ATP, ATP analogues and ATP-competitive inhibitors [40]. In Figure 9, (A) and (C) show how the aeroplysinin-1 compound is bound to the EGFR kinase domain. These results are in accordance with those reported for the Erlotinib drug that is bound to the same binding site in the 1M17 crystallographic structure. Images (B) and (D) show the molecular interactions between aeroplysinin-1 and the amino acids of the EGFR domain kinase cleft. Image (B) shows that the H17 of the -OH second group forms a H-bond with the MET-769 amide oxygen. In the case of the Erlotinib compound complexed with the EGFR kinase domain, the N1 accepts an H-bond from the MET-769 amide nitrogen [40]. Image (D) shows the H-bond created between the O14 and a hydrogen from the side chain of the LYS-721. In the studies with the kinase inhibitor Erlotinib, it was found that the THR-766, LYS-721 and LEU-764 are <4 Å from the acetylene moiety on the anilino ring [40]. The docking results obtained from the SMPSO can explain other enzymatic studies with semi-synthetic derivatives of aeroplysinin-1 that have an inhibitory activity against N+/K+ ATPase [41]. These results can clarify how this compound inhibits the EGFR enzymatic activity and show its possible applicability to targets, like other tyrosine kinase receptors involved in cell proliferation and growth. Furthermore, the multi-objective approach makes the selection of those solutions with different weights of *E_inter_* and *E_intra_* easier, as mentioned.

**Figure 10.**
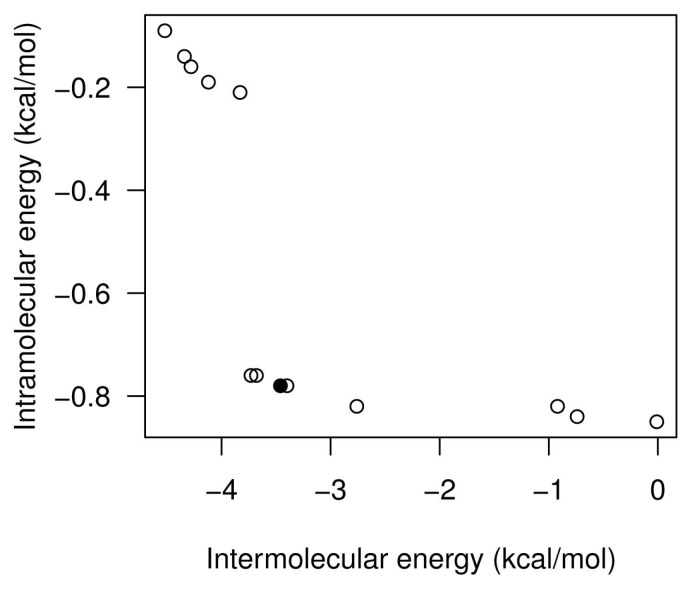
The plot represents all sets of solutions obtained from the SMPSO algorithm from Run 5. The black point corresponds to the solution selected. The final binding energy of the conformation selected equals −2.27 kcal/mol. The *E_inter_* and *E_intra_* values are −3.46 and −0.78 kcal/mol, respectively.

Figure 10 shows the set of non-dominated solutions of the SMPSO algorithm Run 5 of the aeroplysinin-1 and ecto-domain EGFR receptor use case. In this instance, the values of the *E_inter_* and *E_intra_* of the selected solution (see the black point in Figure 10) are −3.46 and −0.78 kcal/mol, respectively. In this case, we have selected a solution not corresponding to the best *E_inter_*, but rather to the solution with a good balance between these two energies. As stated, *E_inter_* represents the binding affinity between ligand-receptor, whereas *E_intra_* describes the energy associated with the ligand deformation in the docking process. In this case, this solution can be interesting given the type of interaction between aeroplysinin-1 and the ecto-domains of the EGFR receptor that is represented and described below.

**Figure 11.**
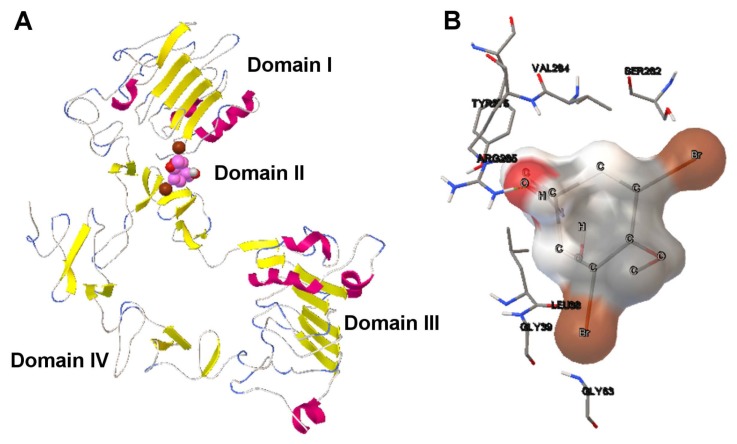
(**A**) The tridimensional structure of EGFR receptor with the four domains labeled; (**B**) The molecular interaction between aeroplysinin-1 and the EGFR receptor. The H-bond is represented by green spheres. The amino acids of the binding site and the ligand atoms have been also labeled.

Image (A) in Figure 11 shows that the aeroplysinin-1 compound of the solution selected is bound to domain II of the EGFR ectodomains. This domain plays an important role in the EGFR receptor activation based on monomer-monomer interactions, as has been reported in previous studies [42]. This mechanism is based on the binding between the EGF (epidermal growth factor) and the EGFR domains I and III, deactivating the EGFR receptor autoinhibition. This conformational change of the receptor leads to the exposition of domains II and IV. Domain II is more involved in the monomer-monomer interaction than domain IV. All of the solutions of the runs preformed by SMPSO have demonstrated that aeroplysinin-1 tends to bind to domain II in terms of the final binding energy. In a more detailed view of the interaction of aeroplysin-1/EGFR ecto-domains, image (B) in Figure 11 shows that an H-bond is formed between the hydrogen of the ARG-285 and H17 of aeroplysinin-1. Such a solution selected with a low *E_intra_* can be useful in cases in which it is necessary to have a more energetic stability of the ligand, like the use case presented in which the aeroplysinin-1 compound interferes in the EGFR dimerization through domain II and requires a more stable docked ligand conformation.

## 5. Experimental Section

In this study, the benchmark of molecular instances used comprises 11 protein-ligand complexes with flexible ligands and receptors. All of these instances have been taken from the PDB (Protein Data Bank) database [43], and they are available online for experimental reproduction. These complexes have been previously selected in [19], where the macromolecule flexibility is included in the energy function of AutoDock 4.2. Table 4 summarizes the set of problems selected, showing the X-ray crystal structures, the PDB accession code and the structure resolution (Å). According to the size and type of ligand, 1D4K, 1HTG, 1HIV and 1HPX complexes include large ligands (the largest being the 1HPX ligand and the smallest 1D4K). The 1HTF complex includes a ligand that is categorized as a small ligand. The 1AJX, 1AJV, 1G2K, 1HVH and 2UPJ complexes include cyclic urea inhibitors. For all instances, according to the indications [19] for the preparation of the benchmark, the torsional degrees of freedom for ligands are limited to 10, selecting those torsions that allow the fewest number of atoms to move around the ligand core. For flexibility in the receptor, the same docking studies performed by [19], demonstrated that residue ARG-8 showed the highest number of bad contacts with the ligand. Therefore, the number of torsional degrees of freedom was limited to six, three torsional degrees for both side-chain of residue ARG-8 of the HIV-protease receptor. The total number of solution variables (*n*) is 23 (3 for translation, 4 for rotation quaternion and 16 for torsional degrees).

In order to solve the docking experiments with AutoDock and jMetal, a set of steps were carried out that consists of the preparation of the ligand and macromolecule, running Autogrid and, finally, running AutoDock and jMetal:

1. Preparation of the ligand and macromolecule: Chimera UCSF software [44] was used to separate the macromolecule and the ligand into two PDB files and to remove small molecules, such as solvent molecules, non-interacting ions, *etc*., from the crystal structure. The AutoDockTools (ADT) graphical interface suite [45] was used to prepare the macromolecule and ligand PDBQT (Protein Data Bank with partial chages and atom type) files. For the preparation of the ligand, partial atomic charges and AutoDock atom types are computed and assigned. For the macromolecule, atom partial charges and hydrogens were added by using the Gasteiger and Babel methods [45]. The rigid and flexible portions of the macromolecule were separately saved as PDBQT files.
2. For running AutoDock, calculation of the grid maps beforehand is necessary in order to reduce the acting area for ligand-macromolecule movements. These maps are calculated by Autogrid, once the coordinates have been established (120 Å ×120 Å ×120 Å) with 0.375 Å of grid spacing. Once Autogrid is executed and the output files obtained, a docking parameter file is configured to run AutoDock.
3. For running AutoDock + jMetal, calculation of the grid maps beforehand is necessary in order to reduce the acting area for ligand-macromolecule movements. These maps are calculated by Autogrid, once the coordinates have been established (120 Å ×120 Å ×120 Å) with 0.375 Å of grid spacing. Once Autogrid is executed and the output files obtained, a docking parameter file is configured to run AutoDock + jMetal.
4. Using the output files created in the previous steps, we have carried out a thorough experimentation consisting of 30 independent runs for each algorithm evaluated (see Section 3) and molecular instance. From the results of these executions, we have calculated the median and interquartile range (IQR) as measures of location (or central tendency) and statistical dispersion, respectively.

**Figure 12.**
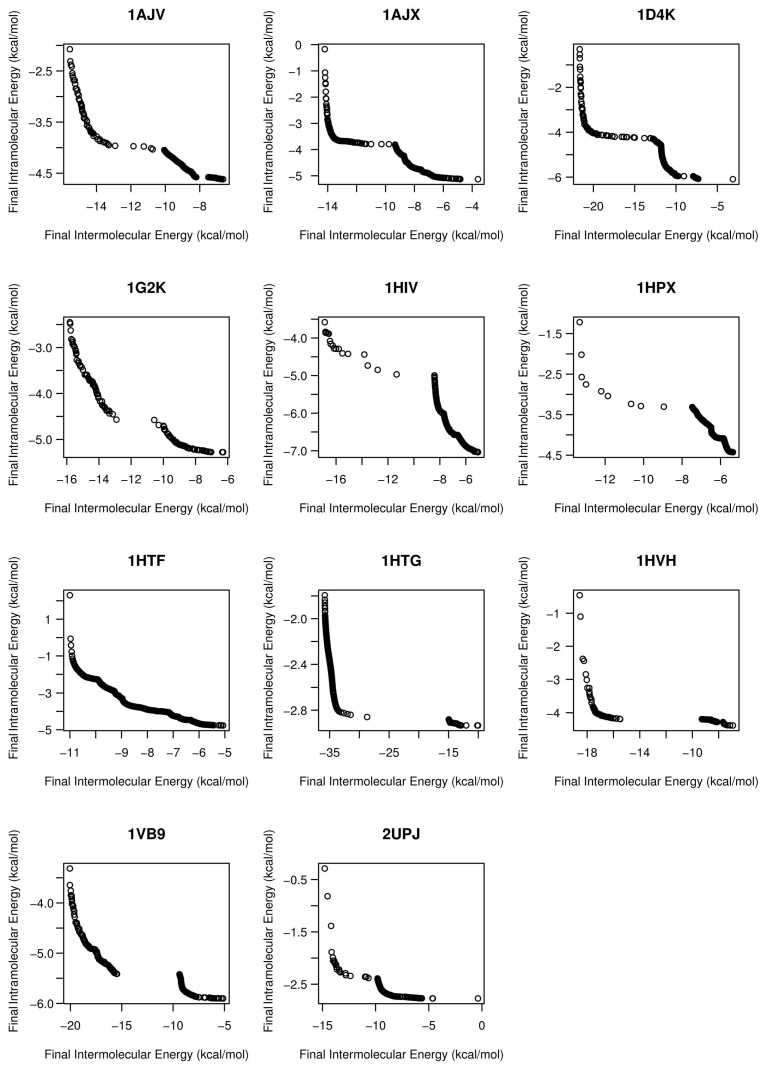
Reference fronts of non-dominated solutions computed from all execution results of all of the evaluated algorithms in this study and for each molecular docking problem instance.

Moreover, to assess the actual performance of the algorithms, we have considered two quality indicators: the hyper-volume (*I_HV_* ) and unary additive epsilon indicators (*I_ɛ_*_+_) [17]. The first indicator takes into account both convergence and diversity, whereas the second one (*I_ɛ_*_+_) is used more to validate the convergence behavior. In this regard, it is worth noting that we are dealing with a real-world optimization problem, and therefore, the true Pareto front to calculate these two metrics is not known. To cope with this issue, we have generated a reference Pareto front for each problem instance, by using all of the non-dominated solutions computed from all execution results of all of the evaluated algorithms in this study. Figure 12 shows the resulting reference fronts for each molecular docking problem instance considered in Table 4, then constituting a common reference to calculate both, *I_HV_* and *I_ɛ_*_+_.

As mentioned, we have used the implementation of the six algorithms studied provided in the jMetalCpp framework (Version 1.5) [18] in combination with AutoDock 4.2 to evaluate the new generated solutions. It is possible to download a prepared version of AutoDock with the jMetalCpp algorithms (along with all of the instances used in this study) in order to reproduce the experiments [46]. To cope with the high computational requirements needed to carry out our experiments, we have used the Condor [47] middleware platform, managing a maximum number of 400 cores, which acts as a distributed task scheduler (each task dealing with one independent run).

### 5.1. Parameter Setup

All of the evaluated algorithms have been configured with a population size of 150 individuals (particles in the case of SMPSO) and a global number of 1,500,000 function evaluations as the stopping condition. These values were selected to be used as the default values by AutoDock algorithms, as in previous studies where the same docking problem instances were tested [48].

For each specific technique, we have used the parameter setup (see Table 5) as recommended in the research study in which the evaluated algorithm was proposed (also used as default parameters in the jMetal framework). In this way, we look to establish comparisons that are as fair as possible between these general purpose algorithms; so no extra parameter refinement has been performed for any technique in particular, and the same context of the preliminary parameter setup is used for all of them.

In particular, for the genetic algorithms NSGA-II and ssNSGA-II, as well as for SMS-EMOA, SBX crossover and polynomial mutation are used as operators for crossover and mutation, respectively. The distribution indexes for these two operators are *η_c_* = 20 for crossover and *η_m_* = 20 for mutation. The crossover probability is *p_c_* = 0.9 and the mutation probability is *p_m_* = 1*=n*, *n* being the number of decision variables of the tackled problem. NSGA-II and ssNSGA-II apply binary tournament selection, while SMS-EMOA uses random selection. In the case of GDE3 (variant rand/1/bin), both mutation constant *μ* and crossover probability *C_r_* take a value of 0.5. In the case of MOEA/D, the mutation constant *μ* is set to 0.5, whereas the crossover probability *C_r_* is 1.0. It also uses a polynomial mutation with values of *η_m_* = 20 and *p_m_* = 1*=n*. The value of the offset for computing the reference point in SMS-EMOA is 20. Finally, for SMPSO, acceleration coefficients ϕ_1_ and ϕ_2_ are set to 1.5; the inertia weight is *W* = 0.9; and a polynomial mutation is applied to one sixth of the particles in the swarm with the same parameters used in the other algorithms.

## 6. Conclusions

The main motivation of this paper has been to carry out a comparative study using six modern multi-objective optimization algorithms and to apply them to the docking problem. The algorithms selected were NSGA-II, ssNSGA-II, SMPSO, GDE3, MOEA/D and SMS-EMOA. A heterogeneous set of 11 protein-ligand complexes with flexible ligands and receptors was selected in order to carry out the experiments.

The benefit of using a multi-objective approach to the molecular docking problem has been confirmed, allowing one to obtain solutions according to the weight of the *E_inter_* and *E_intra_* energies, instead of obtaining just one solution from AutoDock.

In the context of the problems analyzed, the experimentation methodology followed and the chosen parameter settings, we can outline the following conclusions:

1. Using a multi-objective approach to solve the molecular docking problem could lead to a broad set of solutions, which can be selected according to the weight of the *E_inter_* and *E_intra_* energies, instead of only getting one solution from AutoDock.
2. SMPSO provides the best overall performance according to the two quality indicators used and for the studied molecular instances.
3. GDE3 and MOEA/D also show a successful performance in terms of convergence and diversity.
4. For all studied molecular instances, SMPSO converges to the region biased towards the *E_inter_* objective, whereas GDE3 and MOEA/D generate their fronts of non-dominated solutions in a different region to the ones of SMPSO, thereby giving a clue to intramolecular energy optimization.
5. According to the mono-objective AutoDock 4.2 fitness function, the SMPSO algorithm found, in most of the cases, better solutions than the ones obtained by the LGA algorithm. This is a noticeable result, since SMPSO is a general purpose optimization technique in addition to having to distribute its resources to different parts of the Pareto front, whereas LGA is specifically adapted to deal with the molecular docking problem.
6. The analysis of the ligand binding site and the molecular interactions shows a better position for the ligand conformation computed by the SMPSO than the best energy binding solution returned by the LGA according to the obtained RMSD values. Furthermore, the molecular interactions between the ligand conformation obtained by the SMPSO are in accordance with the reference ligand interactions reported in the literature.
7. We have also provided a use case of drug discovery that involves the aeroplysinin-1 compound and the EGFR. The results have demonstrated that according to the use cases presented, it can be more interesting to select a specific docking solution with a balanced tradeoff between *E_inter_* and *E_intra_* values. This approach can improve the way in which the expert can select a solution and contributes to the current methods of drug discovery.

A number of future lines of research can arise using the work presented here as a starting point. The most natural extension would be to design a hybrid algorithm combining search procedures from both the SMPSO and GDE3 algorithms in order to get solutions covering the full Pareto front. A sensitivity analysis of the parameter settings of the studied algorithms could be interesting to reinforce the presented results or to not discard the worst performing algorithms.

Furthermore, a greater number of molecular instances (with a greater number of torsional degrees of freedom for ligands) could be used, and the solutions obtained could be studied from a more biological point of view.

## Figures and Tables

**Table 1.** Median and interquartile range of *I_HV_* *and I_ɛ_*_+_ quality indicators, for each evaluated algorithm and docking instance. Best and second best median results have grey and clearer grey backgrounds, respectively.

*I_HV_*	Hyper-Volume					

	NSGA-II	ssNSGA-II	SMPSO	GDE3	MOEA/D	SMS-EMOA
1AJV	4.75×10^−2^_1.1×10^−1^_	7.47×10^−4^_3.1×10^−2^_	2.65×10^−1^_9.5×10^−2^_	1.23×10^−1^_9.2×10^−2^_	7.39×10^−2^_1.3×10^−1^_	1.33×10^−2^_1.4×10^−1^_
1AJX	2.91×10^−1^_1.2×10^−1^_	2.96×10^−1^_1.3×10^−1^_	5.69×10^−1^_1.1×10^−1^_	3.44×10^−1^_10^−2^_	3.22×10^−1^_1.2×10^−1^_	3.07×10^−1^_1.6×10^−1^_
1BV9	1.33×10^−1^_1.3×10^−1^_	1.17×10^−1^_1.1×10^−1^_	5.41×10^−1^_3.7×10^−1^_	1.79×10^−1^_1.9×10^−2^_	1.81×10^−1^_1.2×10^−1^_	1.34×10^−1^_1.1×10^−1^_
1D4K	2.38×10^−1^_1.3×10^−1^_	2.54×10^−1^_1.2×10^−1^_	5.09×10^−1^_1.5×10^−1^_	3.79×10^−1^_10^−2^_	2.76×10^−1^_7.8×10^−2^_	2.62×10^−1^_9.6×10^−2^_
1G2K	8.06×10^−2^_1.5×10^−1^_	6.11×10^−2^_1.0×10^−1^_	5.74×10^−1^_2.0×10^−1^_	1.62×10^−1^_6.4×10^−2^_	1.13×10^−1^_1.2×10^−1^_	5.69×10^−2^_1.0×10^−1^_
1HIV	7.12×10^−2^_9.0×10^−2^_	5.39×10^−2^_8.0×10^−2^_	7.61×10^−2^_8.5×10^−2^_	1.10×10^−1^_7.8×10^−2^_	7.78×10^−2^_8.8×10^−2^_	5.80×10^−2^_9.5×10^−2^_
1HPX	5.77×10^−2^_1.3×10^−1^_	2.09×10^−2^_6.8×10^−2^_	3.06×10^−1^_3.8×10^−1^_	9.50×10^−2^_4.2×10^−2^_	8.69×10^−2^_9.7×10^−2^_	4.93×10^−2^_1.1×10^−1^_
1HTF	2.80×10^−1^_2.8×10^−1^_	2.85×10^−1^_2.0×10^−1^_	5.29×10^−2^_1.3×10^−1^_	4.03×10^−1^_1.3×10^−1^_	4.98×10^−1^_1.8×10^−1^_	2.95×10^−1^_2.1×10^−1^_
1HTG	9.20×10^−2^_1.0×10^−1^_	9.08×10^−2^_8.5×10^−2^_	3.68×10^−3^_2.7×10^−2^_	1.80×10^−1^_1.6×10^−2^_	1.62×10^−1^_3.3×10^−2^_	1.47×10^−1^_8.8×10^−2^_
1HVH	2.10×10^−1^_1.5×10^−1^_	9.27×10^−2^_1.1×10^−1^_	5.04×10^−1^_2.9×10^−1^_	3.28×10^−1^_1.0×10^−1^_	1.78×10^−1^_1.9×10^−1^_	9.22×10^−2^_2.2×10^−1^_
2UPJ	3.65×10^−1^_7.2×10^−2^_	3.75×10^−1^_9.5×10^−2^_	4.23×10^−1^_1.1×10^−1^_	5.20×10^−1^_1.6×10^−1^_	4.05×10^−1^_9.3×10^−2^_	3.75×10^−1^_1.1×10^−1^_

*I_ɛ_*_+_	Epsilon					

	NSGA-II	ssNSGA-II	SMPSO	GDE3	MOEA/D	SMS-EMOA

1AJV	7.74×10^+0^_2.2×10^+0^_	8.64×10^+0^_1.7×10^+0^_	1.48×10^+0^_3.2×10^−1^_	6.55×10^+0^_8.3×10^−1^_	6.84×10^+0^_1.7×10^+0^_	8.21×10^+0^_2.4×10^+0^_
1AJX	6.65×10^+0^_1.5×10^+0^_	7.04×10^+0^_1.4×10^+0^_	1.64×10^+0^_2.6×10^−1^_	6.44×10^+0^_3.9×10^−1^_	6.18×10^+0^_9.5×10^−1^_	6.51×10^+0^_2.0×10^+0^_
1BV9	1.11×10^+1^_1.8×10^+0^_	1.15×10^+1^_2.0×10^+0^_	1.09×10^+0^_8.7×10^−1^_	1.15×10^+1^_3.7×10^−1^_	1.03×10^+1^_1.6×10^+0^_	1.15×10^+1^_2.0×10^+0^_
1D4K	1.15×10^+1^_2.5×10^+0^_	1.17×10^+1^_2.4×10^+0^_	2.45×10^+0^_6.7×10^−1^_	1.06×10^+1^_1.3×10^+0^_	1.11×10^+1^_1.7×10^+0^_	1.14×10^+1^_2.5×10^+0^_
1G2K	7.55×10^+0^_2.4×10^+0^_	7.81×10^+0^_1.7×10^+0^_	9.30×10^+0^_5.1×10^−1^_	6.13×10^+0^_1.0×10^+0^_	6.99×10^+0^_1.7×10^+0^_	7.88×10^+0^_1.8×10^+0^_
1HIV	8.85×10^+0^_1.7×10^+0^_	9.49×10^+0^_1.5×10^+0^_	3.11×10^+0^_3.4×10^+0^_	8.75×10^+0^_1.2×10^+0^_	7.88×10^+0^_1.8×10^+0^_	9.36×10^+0^_2.0×10^+0^_
1HPX	6.98×10^+0^_1.7×10^+0^_	7.28×10^+0^_1.2×10^+0^_	2.60×10^+0^_6.0×10^+0^_	6.93×10^+0^_3.6×10^−1^_	6.04×10^+0^_1.4×10^+0^_	7.18×10^+0^_1.2×10^+0^_
1HTF	5.26×10^+0^_2.0×10^+0^_	5.23×10^+0^_1.5×10^+0^_	6.93×10^+0^_1.4×10^+0^_	4.37×10^+0^_9.4×10^−1^_	3.65×10^+0^_1.3×10^+0^_	5.16×10^+0^_1.6×10^+0^_
1HTG	2.23×10^+1^_2.9×10^+0^_	2.23×10^+1^_2.3×10^+0^_	3.57×10^+0^_3.8×10^+0^_	1.97×10^+1^_8.8×10^−1^_	1.86×10^+1^_1.2×10^+0^_	2.09×10^+1^_2.6×10^+0^_
1HVH	8.56×10^+0^_2.5×10^+0^_	9.92×10^+0^_2.1×10^+0^_	3.08×10^+0^_2.2×10^+0^_	7.54×10^+0^_1.2×10^+0^_	7.53×10^+0^_2.2×10^+0^_	1.01×10^+1^_3.3×10^+0^_
2UPJ	8.05×10^+0^_1.6×10^+0^_	7.74×10^+0^_2.2×10^+0^_	1.60×10^+0^_2.2×10^−1^_	5.82×10^+0^_2.7×10^+0^_	6.45×10^+0^_1.0×10^+0^_	7.59×10^+0^_1.8×10^+0^_

**Table 2.** Average Friedman’s rankings with Holm’s adjusted *p*-values (*α* = 0.05) of the compared algorithms (SMPSO, GDE3, MOEA/D, SMS-EMOA, ssNSGA-II and NSGA-II) for the test set of 11 docking instances.

Hyper-Volume (*I_HV_* )			Epsilon (*I_ɛ_*_+_)			Metrics’ Hits	

Algorithm	*Fri_Rank_*	*Holm_Ap_*	Algorithm	*Fri_Rank_*	*Holm_Ap_*	Algorithm	*Ranking*
**GDE3** *	**1.81**	-	**SMPSO** *	**1.45**	-	**SMPSO**	**(2 + 1 = 3)**
SMPSO	2.18	6.48×10^−1^	MOEA/D	2.27	3.05×10^−1^	GDE3	(1 + 3 = 4)
MOEA/D	2.63	6.10×10^−1^	GDE3	2.72	2.21×10^−1^	MOEA/D	(3 + 2 = 5)
SMS-EMOA	4.50	2.32×10^−3^	NSGA-II	4.50	4.04×10^−4^	NSGA-II	(5 + 4 = 9)
NSGA-II	4.63	1.64×10^−3^	SMS-EMOA	4.63	2.65×10^−4^	SMS-EMOA	(6 + 5 = 11)
ssNSGA-II	5.22	9.62×10^−5^	ssNSGA-II	5.40	3.57×10^−5^	ssNSGA-II	(6 + 6 = 12)

* indicates the control algorithm, and the column at the right contains the overall ranking of positions with regards to *I_HV_* *and I_ɛ_*_+_.

**Table 3.** Best binding energy values (kcal/mol) calculated from all SMPSO solutions in comparison with the best values of LGA mono-objective solutions. The grey background represents the best solutions in terms of binding energy.

Algorithms/Instance	1AJV	1AJX	1BV9	1D4K	1G2K	1HIV	1HPX	1HTF	1HTG	1HVH	2UPJ
SMPSO	−12.56	−11.22	−17.07	−18.67	−12.83	−13.89	−10.34	−6.83	−31.66	−15.59	−10.91
LGA	−7.26	−6.20	−7.62	−11.25	−7.19	−9.06	−5.70	−7.30	−31.79	−9.39	−5.90

**Table 4.** X-ray crystal structure coordinates taken from PDB database. Their accession codes from the PDB database and the resolution are also presented.

Protein-Ligand Complexes	PDB Code	Resolution (Å)
HIV-1 protease/AHA006	1AJV	2
HIV-1 protease/AHA001	1AJX	2
HIV-1 protease/*α*-D-glucose	1BV9	2.20
HIV-1 protease/macrocyclic peptidomimetic inhibitor 8	1D4K	1.85
HIV-1 protease/AHA047	1G2K	1.95
HIV-1 protease/U75875	1HIV	2
HIV-1 protease/KNI-272	1HPX	2
HIV-1 protease/GR126045	1HTF	2.20
HIV-1 protease/GR137615	1HTG	2
HIV-1 protease/Q8261	1HVH	1.80
HIV-1 protease/U100313	2UPJ	3

**Table 5.** Parameter settings of the compared algorithms. NSGA-II, non-dominated sorting genetic algorithm II; ssNSGA-II, steady-state NSGA-II; SMPSO, speed modulation multi-objective particle swarm optimization; GDE3, third evolution step of generalized differential evolution; MOEA/D, multi-objective evolutionary algorithm based on decomposition; SMS-EMOA, S-metric evolutionary multi-objective optimization.

Algorithm	Parameter	Value
NSGA-II	Selection	Binary Tournament
ssNSGA-II	Crossover	SBX (*p_c_* = 0.9; *η_c_* = 20)
	Mutation	Polynomial (*p_m_* = 1*=n*; *η_m_* = 20)

SMPSO	Archive size	150
	Acceleration coefficients	ϕ_1_ = 1, 5; ϕ_2_ = 1, 5
	Inertia	*W* = 0.9
	Mutation	Polynomial (*p_m_* = 1*=n*; *η_m_* = 20)

GDE3	DE variant	rand/1/bin
	Mutation	*μ* = 0.5
	Crossover	*C_r_* = 0.5

MOEA/D	DE variant	rand/1/bin
	Crossover	*C_r_* = 1.0
	Mutation	*μ* = 0.5; Polynomial (*p_m_* = 1*=n*; *η_m_* = 20)

SMS-EMOA	Selection	Random
	Crossover	SBX (*p_c_* = 0.9; *η_c_* = 20)
	Mutation	Polynomial (*p_m_* = 1*=n*; *η_m_* = 20)
	Reference point	Maximum objective values plus offset=20

*Common parameters*	Population size	150 individuals (or particles)
	Stopping condition	1,500,000 function evaluations
